# Investigating the effect of adding comparisons with prior mammograms to standalone digital breast tomosynthesis screening

**DOI:** 10.1117/1.JMI.12.S2.S22003

**Published:** 2025-03-24

**Authors:** Pontus Timberg, Gustav Hellgren, Magnus Dustler, Anders Tingberg

**Affiliations:** a Lund University, Medical Radiation Physics, Department of Translational Medicine, Faculty of Medicine, Malmö, Sweden; b Skåne University Hospital, Radiation Physics, Department of Hematology, Oncology and Radiation Physics, Malmö, Sweden; c Lund University, Diagnostic Radiology, Department of Translational Medicine, Faculty of Medicine, Malmö, Sweden

**Keywords:** digital breast tomosynthesis, digital mammography, synthetic mammography

## Abstract

**Purpose:**

The purpose is to retrospectively investigate how the addition of prior and concurrent mammograms affects wide-angle digital breast tomosynthesis (DBT) screening false-positive recall rates, malignancy scoring, and recall agreement.

**Approach:**

A total of 200 cases were selected from the Malmö Breast Tomosynthesis Screening Trial. They consist of 150 recalled cases [30 true positives (TPs), 120 false positives (FPs), and 50 healthy, non-recalled true-negative (TN) cases]. The positive cases were categorized based on being recalled by either DBT, digital mammography (DM), or both. Each case had DBT, synthetic mammography (SM), and DM (prior screening round) images. Five radiologists participated in a reading study where detection, risk of malignancy, and recall were assessed. They read each case twice, once using only DBT and once using DBT together with SM and DM priors.

**Results:**

The results showed a significant reduction in recall rates for all FP categories, as well as for the TN cases, when adding SM and prior DM to DBT. This resulted also in a significant increase in recall agreement for these categories, with more of the negative cases being recalled by few or no readers. These categories were overall rated as appearing more malignant in the DBT reading arm. For the TP categories, there was a significant decrease in recalls for DM-recalled cancers (p=0.047), but no significant difference for DBT-recalled cancers (p=0.063), or DBT/DM-recalled cancers (p=0.208).

**Conclusions:**

Similar to the documented effect of priors in DM screening, we suggest that added two-dimensional priors improve the specificity of DBT screening but may reduce the sensitivity.

## 1 Introduction

In today’s breast cancer screening, the prevalent method is two-view [craniocaudal (CC) and mediolateral oblique (MLO)] full-field digital mammography (FFDM).^1^ One well-known drawback of two-dimensional imaging is overlapping tissue that may hide malignant findings or falsely appear as one. One solution to this issue is digital breast tomosynthesis (DBT)—a pseudo-three-dimensional (3D) imaging technique where the X-ray tube moves in a limited angular span while acquiring projection images of the breast and reconstructing an image volume.^2^ The angular span determines the in-depth resolution of the DBT volume, with a wide-angle (WA) system resulting in a higher in-depth resolution compared with narrow-angle (NA) systems. Studies indicate that DBT increases breast cancer detection rates when used in a screening setting.^3^^,^^4^ These studies mainly focus on using DBT as an adjunct to digital mammography (DM), and not as a standalone modality. Performing both DBT and DM will substantially increase the patient dose and may add extra examination time. Furthermore, a majority of these studies evaluated NA-DBT systems and not WA-DBT.

One exception is the Malmö Breast Tomosynthesis Screening Trial (MBTST) where one-view WA-based DBT (in the MLO projection) was compared with two-view FFDM (MLO+CC).^5^ The results from this study showed that 34% more cancers were detected using DBT alone—indicating that the information in the DBT volumes may be sufficient to increase cancer detection without the added scan time and patient dose from additional DMs or the CC projection with DBT. One drawback with DBT as a standalone modality was an increased false-positive (FP) recall rate compared with DM. This may be the result of uncertainties introduced because of the lack of prior images that can be directly compared with acquired DBT volumes. From the available MBTST reading data, it is not possible to separate the effect of comparing two-dimensional (2D) DM priors with DBT volumes, except that it had a very minor effect on cancer detection (<1%).

In DM screening, the use of priors is important in following changes in breast tissue over time.^6^^,^^7^ Though using current DBTs to compare to prior DBTs is possible, it is time-consuming to simultaneously scroll through and scan two 3D volumes, especially if they have different numbers of slices. Two-dimensional DM images are much easier to quickly compare side by side. Instead of acquiring both DBT and DM for a single examination, DMs can be replaced by 2D synthetic mammograms (SMs) reconstructed from the already acquired DBT volumes.^8^ This technique does not add any additional radiation dose and results in a 2D image with an appearance similar to regular DM. There are several studies where DBT with the addition of either DM or SM are compared, with equivalent results;^9^^,^^10^ however, most of them are focused on SM from NA-DBT systems. One study by Clauser et al. evaluated SM based on WA-DBT. The conclusion was that DM can be replaced by SM as an adjunct to DBT.^11^ One limitation of that study was that DBT+SM never was compared with DBT used as a standalone modality. The MBTST dataset introduces a unique opportunity to conduct such studies, and recently, we published a paper indicating no substantial differences in sensitivity and specificity when comparing DM with SM from a WA-DBT system.^12^

Though, as mentioned, the addition of priors reduces recalls in DM screening, the question remains how they affect DBT screening, especially as DBT is an inherently more sensitive method and detects structures that are not visible on DM.

The purpose of this study was to retrospectively investigate how the addition of prior and concurrent mammograms affects WA-DBT screening false-positive recall rates, malignancy scoring, and recall agreement.

## 2 Material and Methods

In this retrospective reader study, readers were asked to review 200 DBT screening examinations from the MBTST study population,^5^ with and without prior DMs and concurrent SMs for comparison. The study was approved by the local Ethics Committee at Lund University (Official Records No. 2009/770).

All DBT cases had been acquired using a commercially available DBT system (Mammomat Inspiration; Siemens Healthineers, Forchheim, Germany). DBT volumes used in this study were retrospectively reconstructed using EMPIRE and synthetic mammograms using Insight 2D (Siemens Healthineers, Forchheim, Germany). All cases were MLO-view only, as only MLO DBT projections were acquired in the MBTST. Thus, only MLO SM views could be reconstructed. DMs from prior screening rounds were acquired on average 22 months, 95% confidence interval (CI) [21.4, 22.6] prior to the DBT acquisition. The Swedish screening program uses an 18- to 24-month screening interval, depending on age. The mean age of the participants at the time of the MBTST acquisition was 57.3 years, 95% CI [55.9, 58.6]. Women without prior DM examinations were excluded.

As the study focused on the effects on recalls, the study population focused on such cases, consisting of 150 recalls and 50 non-recalled cases (confirmed healthy by at least 2 years follow-up). In total, MBTST included 14,848 women of which 535 were recalled based on DBT and 370 on DM, and of those, 137 had cancers detected at the screening occasion. All included cases were randomly selected from the appropriate subgroup of the whole MBTST population.

**Fig. 1:**
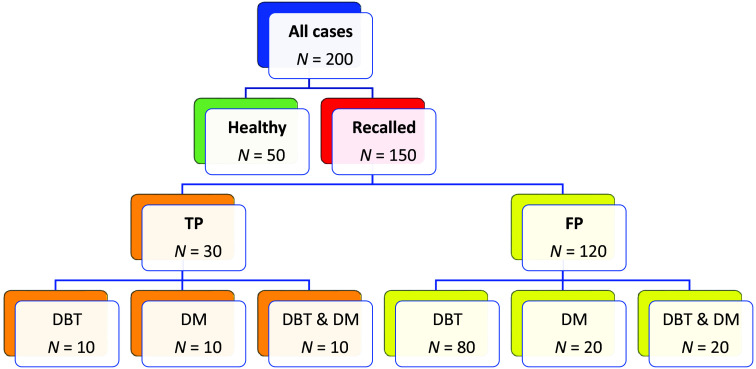
Overview of the cases used in this study. The boxes in the bottom row show which modality was decisive, i.e., if a case was recalled based on DM, DBT, or both in MBTST.

Recalled cases were categorized as either confirmed cancers (n=30) or false positives (n=120). These cases were further split into subcategories depending on the modality on which the recall decision was made: DBT, DM, or DBT/DM (i.e., being recalled on both modalities). These recall decisions were based on the results of the first reader in the MBTST to not bias the reading study due to using double reading as a point of comparison. A majority (n=80) of the recalled cases used in the study consisted of FPs recalled on DBT, but not recalled on DM, because this was the category where SM and priors would most likely be potentially useful. The remaining FP and cancer cases were equally distributed, recalled either only on DM or on both DM and DBT. An overview of the case categories, case distribution, and MBTST correlation is shown in Fig. 1.

Five experienced (>10 years) breast radiologists participated in this reader study. To avoid preference bias, they were not informed that any of the images were synthetic mammograms. They were simply asked to review them as standard DMs and DBT volumes. The only information provided regarding the image material was which image was the prior. Each case was reviewed using ViewDex 2.0,^13^^–^^15^ and during each session, the radiologist was asked to read each image and/or DBT volume and grade the overall risk of malignancy using 10 confidence levels: 1—definitely no malignancy present; 2—low probability of malignancy; 3 to 8—(…); 9—high probability of malignancy; and 10—definitely malignancy present.

The grading was followed by a recall decision as if in a real screening situation (yes/no).

Reading was conducted in two batches. Each batch of cases had two segments so that each radiologist reviewed half of the cases from each study protocol without overlap, i.e., 100 cases reading DBT only followed by 100 cases of reading DBT+SM/DM (prior). To counterbalance the segments the reading order was switched. A training session was carried out before starting the reading of each batch. Each radiologist reviewed a training set consisting of 10 cases and the results from that reading were discarded. There was a washout period of at least 3 weeks between the two reading batches to prevent potential bias.

All readings were performed using high-resolution diagnostic monitors (Barco NV, Kortrijk, Belgium, or Eizo Corp., Hakusan, Ishikawa, Japan) used in the clinic for breast imaging reviews, in a clinically used radiology reading room with adequate ambient light conditions. Readers had no time restrictions and were allowed to split reading a batch into any number of individual reading sessions.

Visual grading characteristic (VGC) statistics were used for the malignancy scoring using VGC Analyzer 1.0.2 to conduct non-parametric, rank-invariant tests similar to receiver operating characteristic (ROC) methodology.^16^^,^^17^ The presented *p*-values were based on fixed-reader analysis using a binormal curve fit, testing for differences in area under the curve (AUC_VGC_).

For the binary recall decisions, the paired two-tailed *t*-test was used to test readers’ observed difference in recall rate for the two reading arms, separately across the different subgroups. To study the recall agreement for each reading arm, the number of readers (0 to 5) recalling each case was compiled for each recall category. In an ideal scenario, all cases in the true-positive (TP) categories should be recalled by all five readers. The opposite goes for the FP and true-negative (TN) categories, where all five readers should agree not to recall any of the cases.

## 3 Results

Both the TN category and all three FP subcategories showed a significant decrease in recall rates in the DBT+SM/prior DM reading arm (p=0.01, p=0.006, p=0.01 and p=0.035). For the TP categories, there was no significant difference in recall rates for either DBT- or DBT/DM-detected cancer cases when SM and priors were added (p=0.063 and p=0.208, respectively), but there was a significant decrease in recalls for the DM-detected cancers in the DBT+SM/prior DM reading arm (p=0.047). Table 1 shows the percentage of cases being recalled averaged for all readers, sorted by case category and reading arm.

**Table 1:** Percentage of cases recalled (by at least three out of five radiologists) and mean malignancy score by case category when studying DBT volumes alone and when combining DBT with SM and the prior DM. P-values are also presented for the difference in the test conditions.

	Recalled cases			Mean malignancy score		
	DBT (%)	DBT + SM/DM (prior) (%)	p-value	DBT	DBT + SM/DM (prior)	p-value
TP DBT (N=10)	78	56	0.063	6.5	5.4	0.06
TP DM (N=10)	36	16	0.047	3.3	2.5	0.15
TP DBT/DM (N=10)	92	86	0,208	7.5	7.3	0.72
FP DBT (N=80)	60	33	0.006	4.5	3.5	<0.01
FP DM (N=20)	39	22	0.01	3.3	3.0	0.02
FP DBT/DM (N=20)	51	35	0.035	3.9	3.4	0.06
TN (N=50)	26	9	0.01	2.7	2.1	<0.01

For the TP categories, our results showed no significant difference in malignancy score between the two reading arms for either recall category (p=0.06, p=0.15 and p=0.72). Regarding the FP and TN recall categories, the malignancy score was significantly higher in the DBT-reading arm for all categories (p<0.2) except for DBT+SM/prior (p=0.06) (Table 1).

In Table 2, the recall agreement is presented. For the TP categories, there was a significantly higher recall agreement in the DBT reading arm due to more cases being recalled by all five readers compared with DBT+SM/DM (prior). For the FP and TN categories, there was a significant increase in recall agreement for the DBT+SM/DM (prior) reading arm, with a clear increase in cases not being recalled by any readers.

**Table 2:** Recall agreement for the combined TP, FP, and TN categories. The total number of cases being recalled by zero to five radiologists is shown for each reading arm (DBT or DBT+SM/DM (prior).

	Number of radiologists recalling each case (0 to 5)					
TP categories (n=30)	0	1	2	3	4	5
DBT	4	3	2	2	5	14
DBT+SM/DM (prior)	7	2	4	7	2	8
FP categories (n=120)	0	1	2	3	4	5
DBT	12	18	24	23	22	21
DBT+SM/DM (prior)	42	24	22	15	8	9
TN category (n=50)	0	1	2	3	4	5
DBT	20	12	11	5	2	0
DBT+SM/DM (prior)	34	11	3	2	0	0

## 4 Discussion

The results of this retrospective reader study, suggest that the addition of 2D priors to WA-DBT (including SM) screening can substantially reduce false-positive recall rates. Our results show a significant decrease in recall rates for FP and TN cases, along with increased recall agreement and a lower overall malignancy score for normal cases.

TP recall rates are difficult to compare among the reading sessions in this study, mainly due to the low number of cases in each subcategory, along with the mixed levels of statistical significance in recall rates and malignancy score. Previously, DM-only recalled cancers significantly decreased in recall rate when priors were added to the assessment, even though the malignancy score showed no significant difference. DM-recalled cancers had an overall low recall rate for both reading arms. It is expected that these cancers would be missed in the DBT reading arm because they were not detected by DBT in the first reading session in the MBTST study. In the current study, the added SM and prior DM actually decreased the recall rate in this category. This may be due to that the readers were asked to always primarily examine the DBT stack and use the SM images mostly for comparison with priors.

The study results agree with older studies on the effect of adding priors to DM screen reading, i.e., a substantial decrease of false positives but no or even negative effect on sensitivity.^6^^,^^7^ For DM priors read together with DBT a study by Kim et al. also showed similar results in that the priors did not positively affect sensitivity.^18^ Essentially, the increase in cancer detection from DBT is a result of that DBT detects cancers that are not visible on 2D mammography, neither DM nor SM. Even if a structure that appears suspicious in 3D seems not to change in 2D, this does not preclude the fact that it has actually progressed and it is simply not possible to see this change on a 2D image.

One thing to note is that twice as many cases with a malignancy score of 4 or lower were recalled in the DBT reading arm (10% versus 5%). The higher recall agreement for the TP categories in the DBT reading arm shows that more cases were uniformly recalled by all five readers. There was, however, an overall mixed recall agreement for the rest of the cases. The FP categories and the TN category showed a clear increase in recall agreement in the DBT+SM/DM (prior) reading arm. This can especially be seen for the FP categories where the agreement is heavily mixed in the DBT reading arm, and the DBT+SM/DM (prior) show a great increase in uniformity with ∼75% of the cases being recalled by two or less readers compared with 45% in the DBT reading arm.

One-view WA-DBT as a standalone modality has already been shown to greatly increase cancer detection compared with DM.^5^ Adding SM should therefore not be seen as a way to further increase cancer detection, but instead as a tool that through providing an efficient way of comparing to prior images can provide greater confidence in whether a case should be recalled or not. Although in this study it seemed to slightly decrease cancer detection compared with only DBT, the positive effect on recalls could counteract this, depending on the desired outcome of the screening program. The results suggest clearly that comparisons with prior images do affect recall decisions, even if these images are acquired with different modalities, but whether this is beneficial or not to screening outcomes is subjective. Adding SMs can be said to bring DBT screening closer to DM screening, though still with substantially higher cancer detection. If 2D images are to be used for quick comparison with priors in a screening situation, it seems reasonable to use SM instead of DM images to save dose and examination time. It should also be noted that although this is a retrospective reading study, the results parallel the difference in outcomes between MBTST as a WA-DBT trial and other NA-DBT trials using SM and/or DM as an adjunct modality for comparison with priors.^19^ MBTST showed a similar cancer detection improvement and somewhat higher recalls, suggesting that the difference may be more an effect of priors than an effect of one-view versus two-view or wide angle versus narrow angle.

Ideally, the study should have been conducted with two rounds of consecutive DBT screening alongside corresponding SM, but we did not have access to such image material for this study. In any case, we see the reading situation as very similar to the one that would be used in such a setup. Two-dimensional images would be used for comparison, and DBT for review.

The study is limited by the relatively small study population. In addition, the absolute recall and detection rates should not be compared with clinical data, both because the set was highly enriched with cancers and contained very difficult “normal,” most of which had been recalled. However, the cases included were highly relevant to the scientific question, as they focus on the specific cases where DBT has been shown to have a positive or negative impact on screening results. Ideally, it would have been interesting to investigate both DBT and SM priors as well, but unfortunately, no such image material was available.

The reconstruction algorithm used in this study for producing the SM images is not the latest version because this was a retrospective study based on an existing image set. Newer versions of the algorithm may influence the results presented in this paper.

## 5 Conclusion

Similar to the documented effect of priors in DM screening, this study suggests that added 2D priors improve the specificity of DBT screening but may reduce the sensitivity.

## Data Availability

Data may be requested by email from the corresponding author. Any access and transfers of data must however fulfill necessary requirements such as local regulatory, ethics, and law.
